# An Innovative Pseudotypes-Based Enzyme-Linked Lectin Assay for the Measurement of Functional Anti-Neuraminidase Antibodies

**DOI:** 10.1371/journal.pone.0135383

**Published:** 2015-08-12

**Authors:** Marua Prevato, Roberta Cozzi, Alfredo Pezzicoli, Anna Rita Taddei, Ilaria Ferlenghi, Avishek Nandi, Emanuele Montomoli, Ethan C. Settembre, Sylvie Bertholet, Alessandra Bonci, Francois Legay

**Affiliations:** 1 University of Siena, Department of Life Sciences, Via A. Moro, 53100, Siena, Italy; 2 GSK, Research Center, Via Fiorentina 1, 53100, Siena, Italy; 3 Section of Electron Microscopy, Great Equipment Center, Tuscia University, 01100, Viterbo, Italy; 4 GSK, Vaccine Research, Holly Springs, North Carolina, 27540, United States of America; 5 GSK, Vaccine Research, Cambridge, Massachusetts, 02139, United States of America; 6 University of Siena, Department of Molecular and Developmental Medicine, Via A. Moro, 53100, Siena, Italy; 7 GSK, Peter Merian Strasse, 4056, Basel, Switzerland; US Food and Drug Administration, UNITED STATES

## Abstract

Antibodies (Ab) to neuraminidase (NA) play a role in limiting influenza infection and might help reduce the disease impact. The most widely used serological assay to measure functional anti-NA immune responses is the Enzyme-Linked Lectin Assay (ELLA) which relies on hemagglutinin (HA) mismatched virus reassortants, or detergent treated viruses as the NA source to overcome interference associated with steric hindrance of anti-HA Ab present in sera. The difficulty in producing and handling these reagents, which are not easily adapted for screening large numbers of samples, limits the routine analysis of functional anti-NA Ab in clinical trials. In this study, we produced influenza lentiviral pseudoparticles (PPs) containing only the NA antigen (NA-PPs) with a simple two-plasmid co-transfection system. NA-PPs were characterized and tested as an innovative source of NA in the NA inhibition (NI) assay. Both swine A/California/07/2009 (H1N1) and avian A/turkey/Turkey/01/2005 (H5N1) N1s within NA-PPs retained their sialidase activity and were specifically inhibited by homologous and N1 subtype-specific, heterologous sheep sera. Moreover, A/California/07/2009 N1-PPs were a better source of NA compared to whole live and detergent treated H1N1 viruses in ELLA, likely due to lack of interference by anti-HA Ab, and absence of possible structural modifications caused by treatment with detergent. This innovative assay is safer and applicable to all NAs. Taken together, these results highlight the potential of NA-PPs-based NI assays to be developed as sensitive, flexible, easy to handle and scalable serological tests for routine NA immune response analysis.

## Introduction

Influenza is an acute viral infection that circulates worldwide, affects any age group and spreads easily from person to person causing often severe illness and death in high risk populations [1]. Influenza viruses belong to the family of *Orthomyxoviridae* and are classified as A, B or C. Influenza A viruses are further divided into subtypes based on the sequence and antigenicity divergence of the two surface glycoproteins, hemagglutinin (HA) and neuraminidase (NA) [2]. HA is responsible for virus attachment to sialic acid-containing receptors on target cells and for internalization and virus-cell fusion [3]. NA contains sialidase activity that contributes to the efficient virus release from infected cells and host spread [4]. In addition, during early stages of viral infection, NA digests decoy receptors that impede the access of virions to respiratory epithelial cells [5].

Vaccination is the best way to prevent influenza and currently available vaccines are designed to elicit an immune response to HA. Anti-HA antibodies (Ab) block virus binding and entry into the host cells, thus neutralizing the virus and preventing infection [2, 6]. Conversely, immunity to NA is considered “infection permissive” [7] since it does not prevent viral infection. Anti-NA Ab hamper virus penetration through the mucinous layer, block the detachment of nascent virions from infected cells, and limit the number of free virions able to infect new cells [8]. Thus, immunity to NA could potentially reduce morbidity and mortality, and limit the opportunity for transmission to other susceptible individuals [9]. NA immunity may prove especially important during a pandemic with a strain carrying a novel HA for which people would be naïve but with an NA for which they may have been primed by previous influenza exposure [4, 8, 10, 11]. Despite these considerations, the amount of NA in vaccine formulations is not standardized, and immune responses to NA are not routinely monitored during the development of influenza vaccines [11] due to the lack of serological assays available for screening large numbers of samples.

The Enzyme-Linked Lectin Assay (ELLA) [12, 13] and the ThioBarbithuric acid Assay (TBA) [11] are the two functional assays most widely used for the specific detection of NA-inhibiting Ab. Both rely on fetuin as the NA substrate but while TBA is based on the chemical conversion of the free sialic acids to a chromogen, ELLA measures the residual terminal galactose exposed after fetuin desialylation using peanut agglutinin. ELLA is preferred to TBA because it shows a far better sensitivity [14] and does not require handling of chemical hazardous reagents. ELLA is a plate-based high throughput assay that allows the testing of a large numbers of samples and is therefore the method of choice for measuring NA-specific antibody responses in animal as well as human studies [15]. HA mismatched virus reassortants [11, 16] and detergent treated viruses [13, 17] have been commonly used as NA sources to overcome interference due to anti-HA Ab present in sera and could impede, by steric hindrance, the access of the substrate into the NA catalytic site [16]. Regardless, these virus alternatives still remain laborious to produce, difficult to handle, and are not optimal for screening large numbers of samples.

The aim of this study was to develop a more practical, safe, and convenient ELLA for the routine analyses of sera, exploiting influenza pseudotyping to generate new sources of NA. Pseudotyped viruses are composed of the structural and enzymatic core of one virus combined with envelope glycoproteins from a second virus, in our case influenza NA [18]. Pseudotyping influenza lentiviral particles is a useful technique that mimics the structure and surface of influenza viruses, which can be used as safer surrogates of pathogenic viruses [19]. Lentiviral HA-containing pseudoparticles (PPs) have been already widely exploited as alternatives to whole live viruses in neutralization assays [20–23].

We demonstrated that lentiviral PPs expressing A/California/07/2009 (H1N1) and A/turkey/Turkey/01/2005 (H5N1) N1s only (NA-PPs) on their surface could be generated. NA-PP preparations were structurally and biochemically characterized, and then tested in ELLA with a set of standard sheep antisera. To demonstrate the superiority of this innovative approach in terms of specificity and sensitivity, HA-specific, NA-specific and HA/NA-specific sera were tested using NA-PPs, with or without a mismatched HA, whole live or detergent-treated viruses.

## Material and Methods

### Materials

Fetuin, carbonate/bicarbonate buffer, Tween 20, Bovine Serum Albumin (BSA), *Clostridium perfrigens* NA, horse radish peroxidase-labelled peanut agglutinin (HRP-PNA), 3,3′,5,5′-Tetramethylbenzidine (TMB), hydrochloric acid (HCl), CelLytic M Reagent, 0.1% Ponceau S, Skim Milk and 2% formaldehyde were bought from Sigma-Aldrich (St. Louis, MO). Sheep polyclonal sera anti-N1 A/California/07/2009 (cat N° 10/218), anti-N1 A/turkey/Turkey/1/2005 (cat N° 8/126), anti-N1 A/NewCaledonia/20/99 (cat N° 4/230), anti-N2 A/Wyoming/3/2003 (cat N° 4/258), anti-NA B/Florida/4/2006 (cat N°. 9/316), anti-NA B/Malaysia/2506/2004 (cat N° 5/252) and pNL4-3luc plasmid encoding Env-defective HIV-1 (cat N° ARP2128) were bought from NIBSC (London, England). HIV-1 p24 protein, rabbit polyclonal anti-HIV-1 p55+p17, and mouse monoclonal anti-HIV-1 p24 antibodies were obtained from Abcam (Cambridge, UK). Dulbecco’s Modified Essential Medium (DMEM), Fetal Bovine Serum (FBS), Penicillin-Streptomycin-Glutamine (PSG), DPBS with Ca^2+^ and Mg^2+^, 4X NuPAGE LDS Sample Buffer, 10X NuPAGE Sample Reducing Agent, NuPAGE Novex 4–12% Bis-Tris Midi Protein Gels, ProLong Gold Antifade Mountant, goat anti-mouse AlexaFluor 568 and goat anti-rabbit AlexaFluor 488 antibodies were purchased from Life Technologies (Carlsbad, CA). Normal Human Serum (NHS) was bought from Lonza (Walkersville, MD). Influenza virus A/California/07/2009 (H1N1) X-181 was kindly provided by Novartis Flu Seed Facility (Novartis Vaccines, Siena, Italy).

Mouse polyclonal sera specific for A/California/07/2009 HA or HA and NA were produced in our lab by immunizing twice intramuscularly 8 Balb/C mice/group with HA-encoding RNA/LNP at the doses of 0.1 μg and 1 μg or with 0.1 μg of MF59-adjuvanted monovalent A/California/07/2009 (H1N1) subunit vaccine, respectively. Pre-immune and Post 2 (15 days after second dose) bleedings were collected for each mouse. Group-pooled sera were inactivated at 56°C for 30 min before antibodies detection by ELLA. All animal studies were carried out in compliance with current Italian legislation on the care and use of animals in experimentation (Legislative Decree 116/92) and with the Novartis Animal Welfare Policy and Standards. Protocols were approved by the Italian Ministry of Health (authorization 249/2011-B) and by the local Novartis Animal Welfare Body (authorization AWB 201106).

### Construction of plasmids expressing NAs

Genes encoding for the full-length sequence of the swine A/California/07/2009 (H1N1) (GenBank accession N° GQ906802.1) and avian A/turkey/Turkey/1/2005 (H5N1) (GenBank accession n° EF619973) NAs were sub-cloned into the ampicillin-resistant pI.18 vector [20] by using *BamH*I and *Xho*I restriction sites. The NA sequence containing an optimal Kozak sequence (RBS) immediately followed by AUG codon was confirmed by sequencing.

### Production of NA-PPs

For pseudotyping, 293T human embryo kidney cells were obtained from American Type Culture Collection (Manassas, VA) and grown in DMEM supplemented with 10% FBS and 1X PSG. Influenza lentiviral pseudoparticles expressing only NA on their surface were generated by co-transfecting 293T cells with a simple two-plasmid system. Briefly, 4 x 10^6^ cells were seeded into 100-mm culture dishes and incubated at 37°C, 5% CO_2_. After 24 h, cells were changed into fresh culture medium containing 5% FBS and were co-transfected with 10 μg of pNL4-3luc plasmid encoding Env-defective HIV-1 and 2 μg of pI.18 encoding A/California/07/2009 (H1N1) (A/CA N1-PPs) or A/turkey/Turkey/01/2005 (H5N1) (A/tk/TK N1-PPs) NA using X-tremeGENE 9 DNA transfection reagent (Roche, Basel, Switzerland). To produce PPs containing a mismatched HA, 2 μg of pI.18 encoding A/Vietnam/1194/04 H5 were added to the transfection mix. 293T cells were transfected with no DNA (Mock) and pI.18 or pNL4-3luc plasmids (ΔN1-PPs) only to produce transfection controls. Supernatants containing N1-PPs were harvested 48 or 72 h post-transfection, cell debris were removed by filtration through a 0.45 μm-pore size filter (Sartorius Stedim Italy S.p.a., Firenze, Italy), divided in aliquots and stored at -80°C. Freeze-thaw cycles were abolished. Sodium dodecyl sulphate-poly-acrylamide gel electrophoresis (SDS-PAGE), western blot (WB) and immunofluorescence were performed to detect NA, HIV-1 capsid (p24) and matrix (p17) proteins expression and localization; transmission electron microscopy (TEM) was used to confirm PPs assembly and NA exposure on their surface; ELLA was used to titrate NA activity and its specific inhibition.

### Western Blotting

Supernatants collected 48–72 h after transfection were mixed with 1X NuPAGE LDS Sample Buffer and 1X NuPAGE Sample Reducing Agent and heated to 100°C for 5 min. Samples were loaded onto NuPAGE Novex 4–12% Bis-Tris Midi Protein Gels and subjected to SDS-PAGE. Protein sizes were estimated using Novex Sharp Pre-stained Protein Standard (MWs: 260, 160, 110, 80, 60, 50, 40, 30, 20, 15, 10, 3.5 kDa) or SeeBlue Prestained Standard markers (MWs: 188, 98, 62, 49, 38, 28, 17, 14, 6, 3 kDa). For WB analysis, proteins were transferred to nitrocellulose membranes using the iBlot 2 Gel Transfer Device (Life technologies). Membranes were first stained with 0.1% Ponceau S solution to verify protein transfer and then were blocked with PBS + 0.1% Tween 20 + 5% milk overnight at 4°C. To detect NA, p24 and p17 proteins, membranes were incubated with sheep polyclonal anti-N1, goat monoclonal anti-p24 and goat polyclonal anti-p55+p17 Ab respectively for 90 min at room temperature. After three washes in PBS + 0.1% Tween 20 (T-PBS) blots were incubated with HRP-labelled donkey anti-sheep, sheep anti-mouse or goat anti-rabbit Ab respectively for 1 h at room temperature. Membranes were washed thrice with T-PBS, twice with PBS and then incubated with SuperSignal West Pico Chemiluminescent Substrate (ECL; Thermo Fisher Scientific, Rockford, IL) for 2 min. In the dark, photographic hyperfilms (GE Healthcare, Rydalmere, NSW, Australia) were placed against the membrane for increasing times and then developed using Curix60 (AGFA HealthCare, Greenville, USA) films processor.

### Indirect immunofluorescent confocal microscopy

Indirect double immunofluorescent staining was used to observe the co-localization of NA and p17 proteins in A/tk/TK N1-PPs, ΔN1-PPs and Mock samples. Culture supernatants were diluted 2-fold in 4% formaldehyde solution and incubated for 20 min on sterile glass slides. After 2 washes with 100 μl of PBS, samples were incubated with rabbit polyclonal anti-HIV-1 p55+p17 and mouse polyclonal anti-NA Ab diluted 1:500 in PBS for 15 min, at room temperature. The slides were washed twice with PBS and incubated with goat anti-rabbit AlexaFluor 488 and goat anti-mouse AlexaFluor 568 Ab, respectively, for 10 min in the dark. Samples were washed and mounted with ProLong Gold Antifade Mountant. Culture supernatants were also incubated with secondary Ab for background detection only. Glass slides were stored at 4°C in the dark before observation with a confocal microscope. Images were obtained using a Zeiss LSM710 (Carl Zeiss, Jena, Germany) equipped with the 63x oil immersion objective and were elaborated with Zen2800 software.

### Transmission electron microscopy

For negative staining experiments droplets of sample suspensions (10 μl) were placed on formvar-carbon coated grids and allowed to adsorb for 60 sec. Excess liquid was removed gently touching the filter paper. The adsorbed specimen was then fixed for 2 min at room temperature floating on a drop of 4% paraformaldehyde in PBS, pH 7.2. Negative-staining was performed by first washing the specimen grid on a drop of negative stain (2% uranyl acetate in distilled water), blotting and repeating this step once more, this time leaving the specimen grid for 60 sec on a new drop of negative stain solution.

For immunogold experiments samples were let to adsorb for 60 sec on nickel formvar-carbon coated grids and the staining was performed on drops of different solutions. No specific sites were blocked with 0.1% BSA in PBS for 15 min and the incubation with anti-NA Ab diluted 1:500 in blocking buffer was carried out for 1 h at room temperature. The grids were washed three times in 0.1% BSA in PBS, 5 min each, and then incubated with a secondary goat anti-mouse Ab conjugated to 5 nm colloidal gold particles (British BioCell International, UK), diluted 1:50 in 0.1% BSA in PBS, for 1 h at room temperature. After rinsing in 0.1% BSA in PBS, the grids were washed briefly with distilled water and negatively stained with 2% uranyl acetate in distilled water. The primary antibody was omitted in control.

Samples were observed at a JEOL 1200 EX II electron microscope. Micrographs were acquired by the Olympus SIS VELETA CCD camera equipped the iTEM software.

### Virus Treatment

The whole live A/California/07/2009 (H1N1) X-181 virus was treated with 1% of Triton X-100 for 1 h at room temperature, in order to distrupt HA while mantaining NA structure and activity [13, 17], to use as a source of NA in ELLA. Then, the detergent was removed from the solution using Pierce Detergent Removal Spin Columns (Thermo Scientific), divided in aliquots, and stored at -80°C avoiding freeze-thaw cycles.

### Enzyme-linked lectin Assay

NA activity and its specific inhibition were assayed using ELLA as previously described [12]. Briefly, Maxisorp Nunc 96 well plates (Thermo Fisher Scientific) were coated with 7.5 μg/well of fetuin and incubated overnight at 4°C. Plates were washed four times with 350 μl/well of PBS + 0.05% Tween 20 (T-PBS), covered and stored for at least 1 month. Duplicates of each culture supernatant or virus were serially diluted in incubation buffer (DPBS containing Ca^2+^, Mg^2+^ and 1% BSA) onto fetuin-coated plates and incubated overnight at 37°C. Serial dilutions of NA from *Clostridium perfrigens* were loaded on the same plate as internal positive control while incubation buffer only was added in two columns for background control. Plates were washed four times with 350 μl/well of T-PBS, and further incubated with 100 μl/well of HRP-labelled peanut agglutinin for 1 h. Plates were washed again and incubated with 100 μl/well of TMB for 30 min at room temperature. The reaction was stopped with 100 μl/well of 0.5 M HCl and absorbance was read at a wavelength of 450 nm using EnVision Multilabel Plate Reader (Perkin Elmer, Waltham, MA, USA). To standardize the amount of NA activity to use in the inhibition assay, a PPs working dilution calculated at OD_450nm_ = 2 and corresponding to the beginning of the linear part of the titration curve, was chosen. Sources of NA (55 μl) were added to 55 μl of heat-inactivated sera, two-fold serially diluted in U-bottom 96-well plates. Eight wells were incubated with 55 μl of NA source only as NA activity positive control while other eight wells were incubated with incubation buffer only for background detection. After 2 h incubation at 37°C, 100 μl from each well were transferred into corresponding wells of a fetuin-coated plate and incubated overnight a 37°C. Plates were washed three times with T-PBS and developed following the same procedure described for the titration of NA activity. Neuraminidase Inhibition (NI) titers were defined as the reciprocal of the serum dilution at which the mean absorbance was ≤ 50% of the mean absorbance measured with the control virus (ID50); samples with a titer < 50 were assigned a value of 25 and an arbitrary cutoff was chosen at a titer = 250 to exclude weak interference given by serum mannan-binding lectins.

### Statistical Analyses

All data were graphed using GraphPad Prism 6.04 software (GraphPad Software, La Jolla, CA, USA) and show mean and standard deviation. Non-parametric, Mann–Whitney test and parametric one-way ANOVA tests were performed on selected groups considering a p-value of 0.05 or less statistically significant. ns: not significant, *** p<0.001, ** p<0.01, * p<0.05.

## Results

### Generation and characterization of N1-PPs

**Fig 1:**
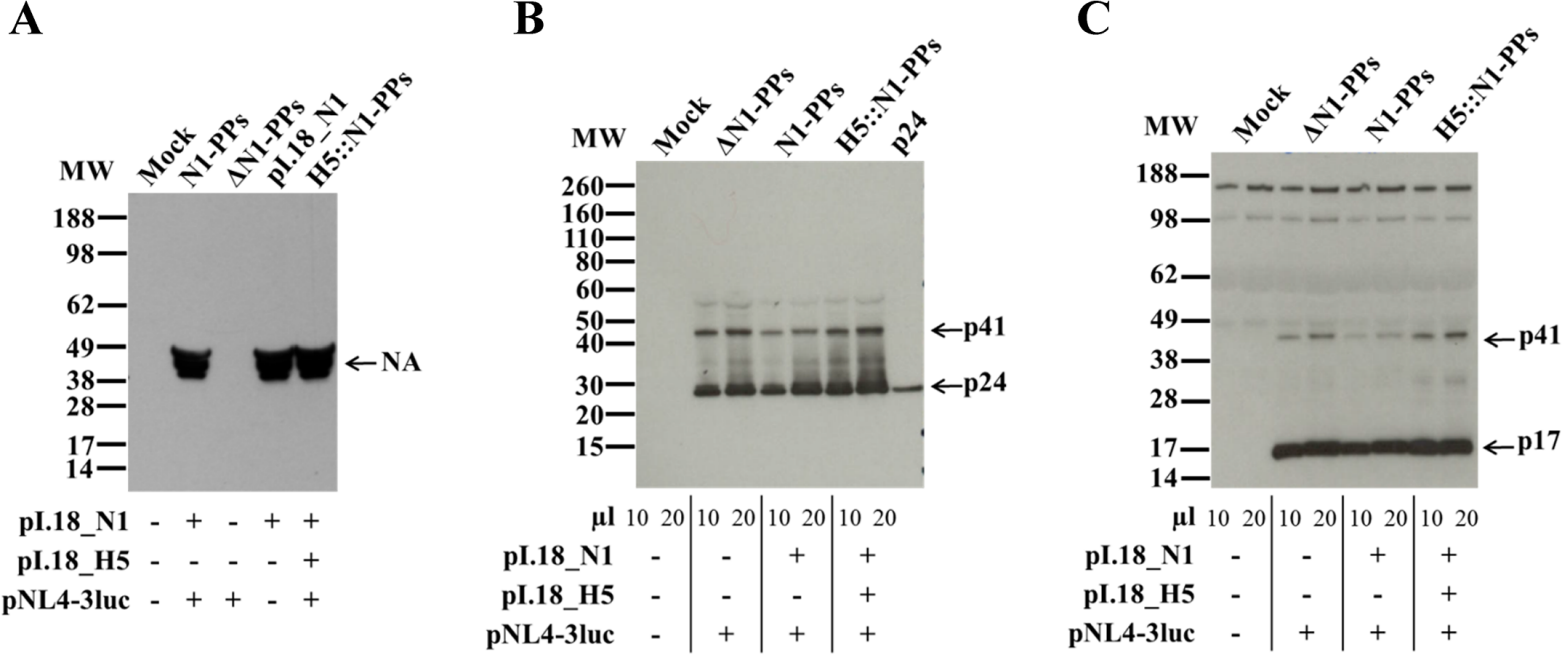
Detection of NA, p24 and p17 proteins in A/tk/TK N1-PPs. Culture supernatants of 293T cells transfected without plasmids (Mock) and with different combinations of the two plasmids (ΔN1-PPs or pI.18_N1) were resolved by 4–12% SDS-PAGE in reducing conditions, transferred to nitrocellulose membrane and immunoblotted with (A) sheep polyclonal anti-N1 A/Turkey, (B) mouse monoclonal anti-p24 and, (C) rabbit polyclonal anti-p55+p17 Ab. Arrows identify the corresponding protein. Data shown are representative of two independent experiments.

Lentiviral pseudoparticles with Influenza A/California/07/2009 or A/turkey/Turkey/01/2005 N1s (N1-PPs) were generated by co-transfection of plasmids encoding for N1 (pI.18_N1) and for Env-defective HIV-1 (pNL4-3luc) in 293T cells. Transfections with no DNA (Mock) and with different combinations of the two plasmids (pI.18_N1 or ΔN1-PPs) were also performed. Culture supernatants were collected 48–72 h post-transfection and the presence of N1 was confirmed by SDS-PAGE in reducing conditions followed by WB using a sheep polyclonal anti-N1 antibody (Fig 1). Bands corresponding to monomeric N1, running at approximately 49 kDa, were detected in supernatants of cells that received the pI.18 plasmid coding for A/tk/TK N1; no differences in N1 band intensity were observed when N1 was expressed alone or in PPs, or in presence or absence of H5 (Fig 1A). To demonstrate that the PPs lentiviral backbone was also produced, the expression of HIV-1 matrix and capsid proteins (p17 and p24, respectively) was characterized. Bands corresponding to the p17 and p24 proteins, together with their p41 precursor, were detected in all supernatants of cells transfected with at least the pNL4-3luc plasmid, at ≈17, ≈24 and ≈41 kDa respectively (Fig 1B–1C).

**Fig 2:**
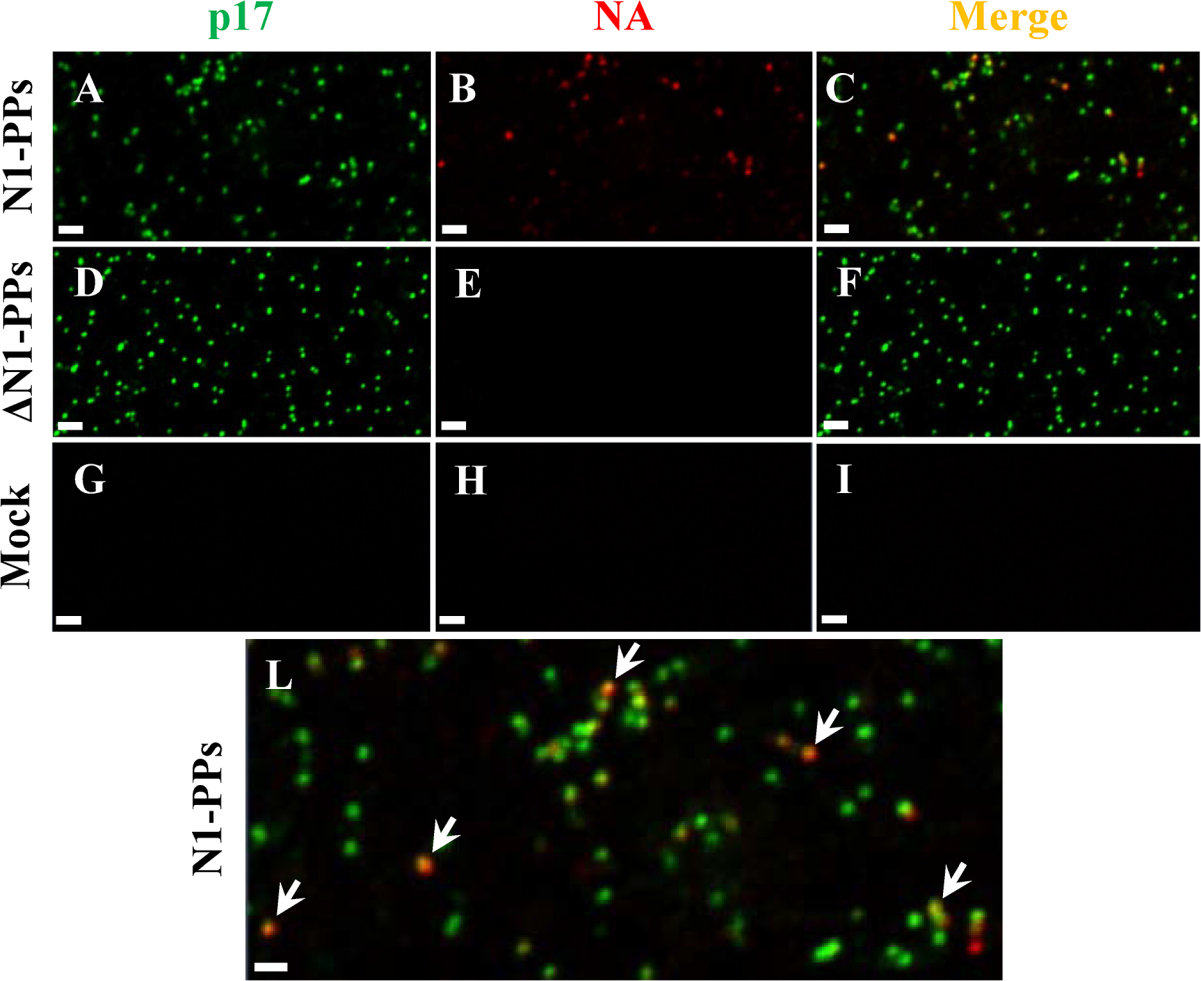
Immunofluorescence analysis of N1-PPs. Culture supernatants of 293T cells transfected with with both pNL4-3luc and pI.18_ N1 from the A/turkey/Turkey/01/2005 influenza strain (A/tk/TK N1-PPs) plasmids, were collected 48 h post-transfection. Confocal microscopy images of N1-PPs (A-B-C), ΔN1-PPs (D-E-F) and Mock (G-H-I) samples stained for N1 (B-E-H) and p17 (A-D-G) proteins with sheep polyclonal anti-N1 A/tk/TK (red) and rabbit polyclonal anti-p55+p17 sera (green), respectively. Fluorescence merges (C-F-I). (L) Magnification of image C. Arrows indicate the co-localization of NA and p17 proteins in N1-PPs sample. Scale bar is 1 μm. Data shown are representative of two independent experiments.

Immunofluorescence experiments were then performed to demonstrate the co-localized expression of p17 and N1 proteins in PPs. PPs, with or without N1, and Mock samples were fixed, double stained, and observed at the confocal microscope. No fluorescence was observed in the Mock sample (Fig 2G–2H), green signals specific for p17 matrix protein were detected in both PPs samples (Fig 2A–2D), while red signals specific for A/tk/TK N1 were found in N1-containing PPs sample only (Fig 2B). Very small spherical particles with a regular shape and homogeneously distributed were observed. Superimposition of p17 and NA fluorescences revealed that not all the particles contained NA and that the amount of NA could change between particles (Fig 2C–2L).

**Fig 3:**
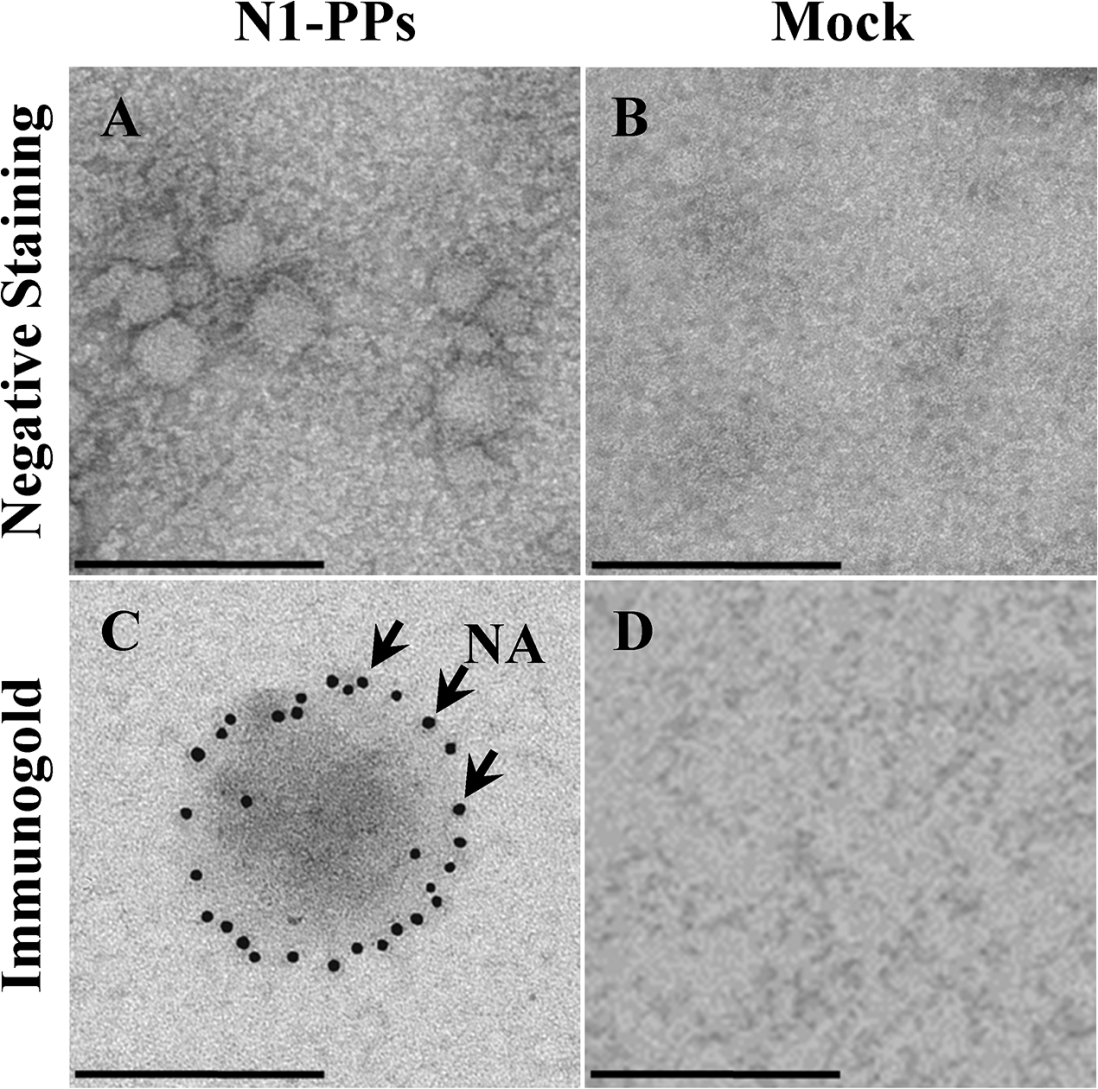
Transmission electron microscopy analysis of N1-PPs. Culture supernatants of 293T cells transfected with no DNA (Mock) and with both pNL4-3luc and pI.18_ N1 from the A/turkey/Turkey/01/2005 influenza strain (A/tk/TK N1-PPs) plasmids, 48 h post-transfection. Negative staining of A/tk/TK N1-PPs (A) and Mock (B), and immunogold labeling of NA in A/tk/TK N1-PPs (C) and Mock (D) samples with mouse polyclonal anti-N1 A/turkey/Turkey/01/2005 serum. Arrows represent gold-labeled Ab bound to NA. Scale bar is 200 nm.

Further evidence of the assembly of pseudoparticles expressing NA was achieved by electron microscopy. Immunogold labelling of NA was performed on A/tk/TK N1-PPs and Mock samples using a specific anti-N1 Ab. Spherical particles with a diameter ranging approximately 50–120 nm were observed (Fig 3A) and gold dots all around the particles confirmed NA localization on the surface (Fig 3C). Meanwhile, no typical viral morphology was found in supernatant of Mock-transfected cells (Fig 3B–3D).

**Fig 4:**
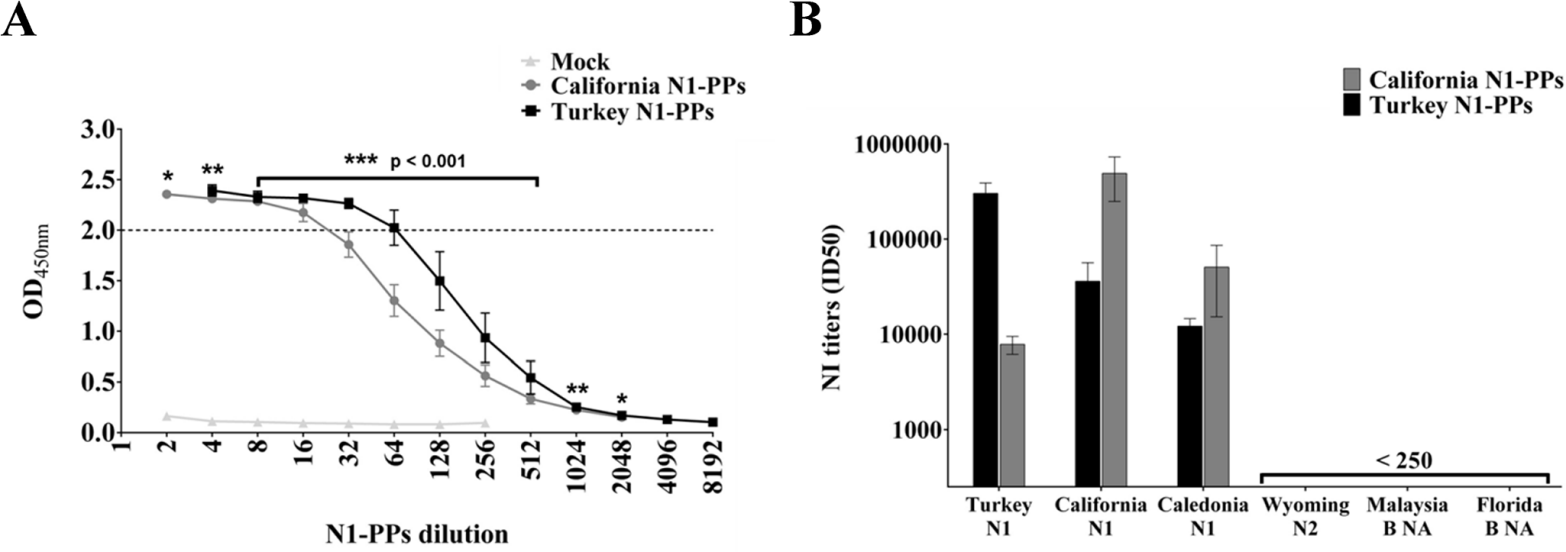
Titration of NA activity and its specific inhibition by ELLA. (A) NA activity in A/CA and A/tk/TK N1-PPs, and Mock samples was titrated incubating fetuin-coated plates with serial dilutions of the culture supernatants. A NA working dilution corresponding to the beginning of the linear part of the titration curve (OD_450nm_ = 2) was selected to standardize NA activity in the inhibition test. Each data point represents the mean and the standard deviation from at least two independent experiments. Non-parametric Mann-Whitney test was performed to compare ODs values at each PPs dilution; *** p < 0.001, ** p < 0.005, and * p < 0.05. (B) Inhibition of A/CA and A/tk/TK N1-PPs enzymatic activity by sera specific for N1 (A/California/07/2009, A/turkey/Turkey/01/2005, A/NewCaledonia/20/99), N2 (A/Wyoming/3/2003) and B (B/Malaysia/2506/2004 and B/Florida/4/2006) NAs. Each bar represents the mean and the standard deviation from at least two independent experiments.

To determine whether N1 in PPs retained enzymatic activity and could be used in ELLA, culture supernatants containing A/CA and A/tk/TK N1-PPs were serially diluted and incubated onto fetuin-coated plates overnight at 37°C. At each dilution of the linear part of the titration curve, the ODs of both A/tk/TK and A/CA N1-PPs were compared. ODs for A/tk/TK N1-PPs were statistically higher than ODs for A/CA N1-PPs, suggesting that A/tk/TK N1-PPs were more active (Fig 4A). No activity was measured in supernatants of Mock controls.

### NA-PPs-based ELLA

Having demonstrated that PP incorporated NA was functional, A/CA and A/tk/TK N1-PPs were investigated for their suitability as an alternative NA source in the ELLA NA inhibition test. To standardize the assay: the amount of NA activity was chosen as a PPs working dilution giving in ELLA an OD_450nm_ = 2. This signal is corresponding to the beginning of the linear part of the titration curve. A/CA and A/tk/TK N1-PPs were diluted 1:25 and 1:60, respectively (Fig 4A), and were incubated with serial dilutions of a panel of sheep sera specific for different NAs. NI titers were expressed as the reciprocal of the serum dilution at which the mean absorbance was ≤ 50% of the mean signal obtained with PPs only control (ID50). As shown in Fig 4B, both A/CA and A/tk/TK N1-PPs were specifically inhibited by homologous and heterologous sera specific for N1 subtypes (A/California/07/2009, A/turkey/Turkey/01/2005 and A/NewCaledonia/20/99), while no cross-neutralizing Ab were detected in sera raised against non-related NAs such as A/Wyoming/3/2003 (H3N2), B/Malaysia/2506/2004 NA and B/Florida/4/2006 NA (Influenza B).

**Fig 5:**
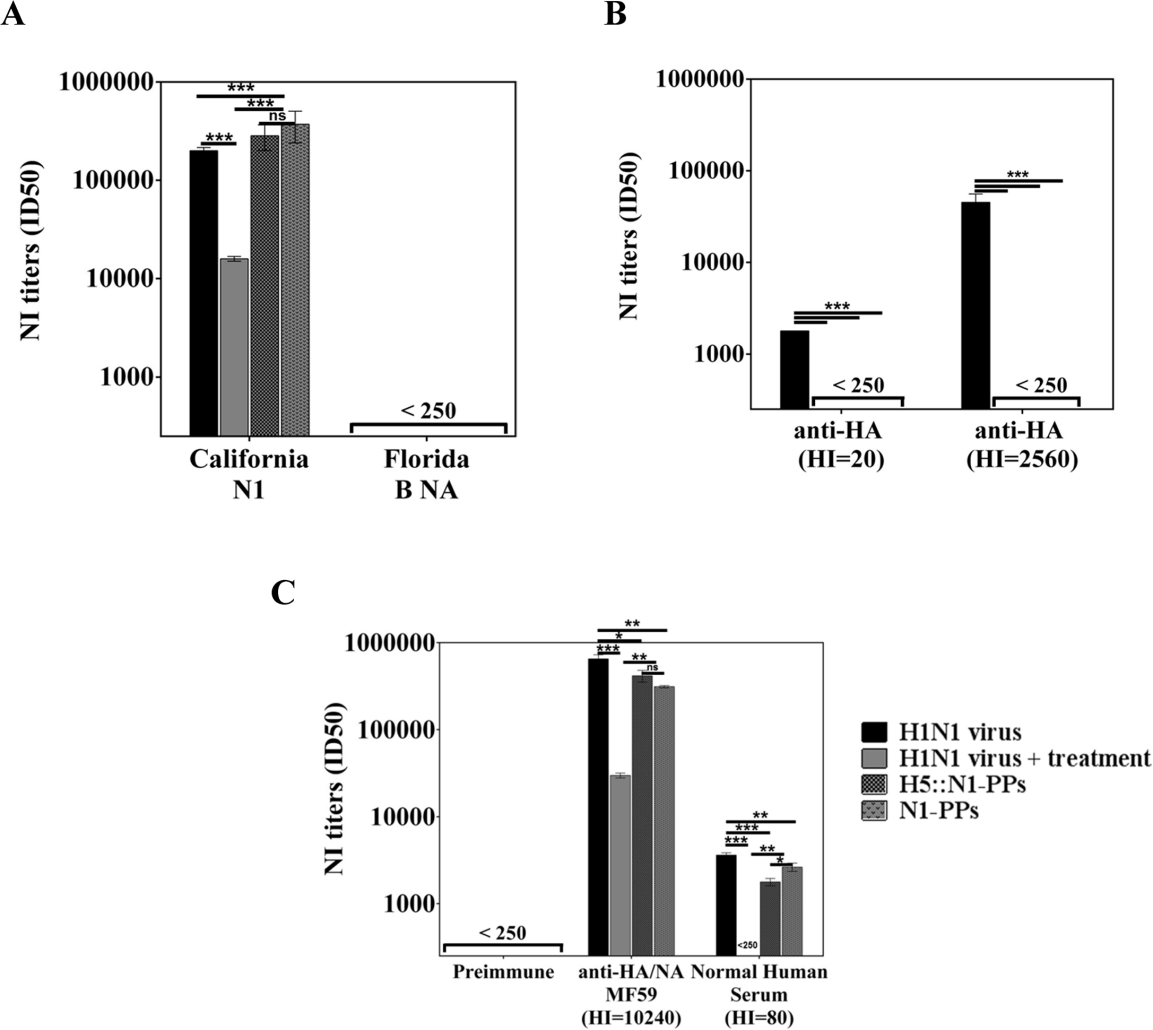
NI titers measured in a panel of sera using different sources N1 from A/California/07/2009 (H1N1) virus. (A) Sheep polyclonal anti-N1 A/California/07/2009 and anti-NA B/Florida/04/2006 sera, (B) mouse polyclonal anti-HA sera with low and high HI titers, and (C) mouse polyclonal preimmune and anti-A/California/07/2009 subunit vaccine sera and normal human serum were tested *vs* the whole live and Triton X-100 treated A/California/07/2009 (H1N1) viruses and *vs* N1-PPs, with or without the mismatched HA from A/Vietnam/1194/04 (H5N1) influenza virus. Each bar represents the mean and the standard deviation from at least two independent experiments. Parametric One-Way ANOVA test was performed to compare NI titers; *** p < 0.001, ** p < 0.005, and * p < 0.05.

To demonstrate that this innovative NA-PPs-based ELLA performs better in terms of specificity and sensitivity than the conventional ELLA based on mismatched HA reassortants or detergent treated viruses, NI titers were measured in a panel of sera raised against HA and/or NA, using different sources of NA. Sheep anti-N1 A/California/07/2009 and anti-NA B/Florida/4/2006 polyclonal sera were chosen to demonstrate assay specificity, while two mouse polyclonal sera specific for A/California/07/2009 HA with low and high HI titers were chosen to investigate anti-HA Ab interference. Finally, sera from mice immunized with MF59-adjuvanted monovalent A/California/07/2009 subunit vaccine, containing both NA and HA, and human normal sera (NHS) were also tested (Fig 5).

Enzymatic activities in all four different sources of NA were specifically inhibited by a homologous serum raised against A/CA N1, while no inhibition was measured with a serum raised against B/Florida/04/2006 NA (Fig 5A). NI titers obtained are significantly greater than those obtained using detergent-treated H1N1 virus. In addition, no NI titers were measured in sera from mice immunized with A/CA HA alone when using NA-PPs, with or without a mismatched HA, while a positive signal was observed when using live A/CA H1N1 virus, suggesting anti-HA Ab interference (Fig 5B). Finally, functional anti-NA Ab were detected by ELLA in normal human sera, and in sera from animals immunized with MF59 adjuvanted A/California/07/2009 H1N1 subunit vaccine. The NI titers obtained using NA-PPs, with or without a mismatched H5 and live virus were comparable, and were significantly higher than those detected using detergent-treated virus (Fig 5C). On the basis of what is demonstrated in Fig 5B the NI titers measured using NA-PPs are clearly due to anti-NA antibodies only. While the NI titer measured using the A/CA H1N1 virus are due to both true specific anti-NA Abs and also to anti-HA Abs interfering by steric hindrance to NA activity. The same consideration now could be retrospectively done for the NI titers measured using the A/CA H1N1 virus in the homologous serum raised against A/CA N1. In addition no NI titers were detected in preimmune sera.

## Discussion

During the recent decades there has been a growing interest in including influenza NA antigen in vaccine design for the purposes of expand strain coverage and increase overall vaccine immunogenicity [2]. To characterize immune responses to NA and their contribution to protection against disease, a first generation of functional serological assays were developed relying on mismatched HA-reassortants or detergent-inactivated viruses. However, with future vaccination strategies that may include standardized amounts of NA in influenza vaccine formulations, it is necessary to implement a simple and safe standard second generation NI assay for the high-throughput screening of large numbers of sera to support vaccine development. In this study, we used a simple two-plasmid transfection system to generate N1 lentiviral PPs. Confocal and electron microscopy revealed that N1-PPs assembled, were spherical, heterogeneous in dimension, and expressed NA on their surface. Both A/California/07/2009 and A/turkey/Turkey/01/2005 N1-PPs showed enzymatic activity that was specifically inhibited by homologous and by cross-reactive anti-NA Ab present in sheep, mouse, and human sera. N1-PPs were more sensitive than detergent-treated influenza virus in detecting NA-specific Ab, and were not affected by the presence of anti-HA Ab. Therefore, we propose NA-PPs as a safe, simple, and innovative source of NA suitable for the measurement of NI titers by ELLA.

Reassortants viruses containing the NA of interest and a mismatched HA [11, 16], as well as detergent-treated viruses [17] are widely used as sources of NA in ELLA, however, the procedures required to produce such reagents are laborious, time-consuming, expensive, and may require biosafety level 2 or higher when highly pathogenic viruses are used [24, 25].

As reported by Subbarao et al. (2003) [26], the production of HA-mismatched reassortant viruses involves the cloning of HA and NA genes of interest and other six RNA segments coming from a different influenza strain, transfecting mammalian cells with plasmids, and finally harvesting, purifying and expanding the viral particles produced. Time and efforts must be spent to choose the most appropriate mismatched HA and to better understand the safety requirements; as a consequence, this would not be practical when multiple reassortants involving different NAs have to be generated. HAs from non-human strains of influenza virus were used to increase the safety of the system but, given that NA also contributes to viral replication and pathogenicity, the presence of an avian N1, for example, could increase the infectivity of the reassortants virus. Chemically inactivated reverse genetics viruses were also used but the approach implied additional experimental work to an already long procedure [11].

The treatment of viruses with detergents, like Triton X-100, was tested to disrupt HA antigen and to reduce virus infectivity but, as for HA-mismatched reassortants viruses, this approach suffers from several limits. First, live viruses are difficult to obtain, may grow poorly, and require higher biosafety level if they are highly pathogenic. Second, even if the procedure is quite easy, incubation with and removal of detergents by columns or beads [13] slow down the procedure and may result in a decreased yield. Finally, detergent treatment might modify the NA structure, hampering Ab access while still retaining some sialidase activity, resulting in an underestimation of NI titers. Furthermore, when viruses are treated with detergents, the viral particles are disrupted and antigens are presented in micelles of detergents that are not representative of the natural viral surface.

In contrast, we demonstrated that enzymatically active N1-PPs, with N1 of either swine or avian origin, were rapidly produced 48–72 h after cell transfection, with a commercial *env*-defective HIV-1-encoding plasmid and an N1-expressing vector. Enough N1-PPs of swine or avian origin were produced in a single 8 ml transfection to assay 400 and 700 sera, respectively. NA-PPs-containing culture supernatants were functional in the ELLA assay and had two key advantages: 1) NA-PPs did not require the co-expression of HA for functionality, and 2) NA enzymatic activity was not affected by the interference of nonspecific anti-HA Ab. Without HA, NA-PPs become a non-infectious reagent that can be handled in a standard laboratory.

Another important aspect to take into account when measuring anti-NA Ab titers, is the interference by anti-HA Ab resulting in false positive NI titers. Animal and human sera may contain anti-HA Ab that bind to matched or highly conserved HA abundantly expressed on the viral surface. Treating the whole virus with detergents disrupts HA that become poorly recognized by anti-HA Ab, while preserving NA activity. In this study, we confirmed the observations by others demonstrating the suitability of this approach to characterize serological immune responses toward NA after vaccination [13, 17]. Others, exploiting the lack of pre-existing anti-H6 Ab in humans, used H6 reassortants to avoid interference by anti-HA Ab and specifically detect anti-NA Ab in human samples [11, 27]. Using NA-PPs containing a mismatched H5, we also confirmed the feasibility of the HA-mismatched approach. Most importantly, our data show that we can perform ELLA with NA-PPs devoid of any HA, completely avoiding the problem of anti-HA Ab interference.

Another advantage of pseudotyping is the ability to generate an expression vector library encoding all known NAs, which could be promptly used for the large scale production of lentiviral PPs [19, 28].

We are now further improving the yield of NA-PPs by using different expression vectors and human codon-optimized NA sequences to improve the expression and incorporation of NA in PPs. Finally, we are extending our initial approach to generating and characterizing PPs expressing NA of N2 or B influenza strains as well as other N1 subtypes.

In conclusion, we propose pseudotyping as an innovative approach for the production of a source of NA better than detergent-treated or mismatched reassortant viruses. The NA-PPs-based ELLA represents an attractive simpler and more specific alternative to classical methods assessing the breath of NA-neutralizing antibodies, which is not hampered by biosafety considerations, poor growth of viruses, or lack of availability of reagents and instrumentations. In addition, to providing a robust and reliable tool for serological evaluations of pre-existing and vaccine-induced immune responses to NA, NA-PPs might also serve in drug resistance studies, to screen for new antiviral drugs, and for structural and biochemical NA characterization.
